# Final results from IMPROVE: a randomized, controlled, open-label, two-arm, cross-over phase IV study to determine patients’ preference for everolimus in combination with exemestane or capecitabine in combination with bevacizumab in advanced HR-positive, HER2-negative breast cancer

**DOI:** 10.1186/s12885-020-06747-y

**Published:** 2020-04-06

**Authors:** Thomas Decker, Ulrike Söling, Antje Hahn, Christoph Maintz, Christian Martin Kurbacher, Ursula Vehling-Kaiser, Dagmar Sent, Peter Klare, Volker Hagen, Marco Chiabudini, Julia Falkenstein, Martin Indorf, Eva Runkel, Karin Potthoff

**Affiliations:** 1 Gemeinschaftspraxis für Hämatologie und Onkologie GbR, Elisabethenstrasse 19, 88212 Ravensburg, Germany; 2 Onkologische Gemeinschaftspraxis, Goethestrasse 47, 34119 Kassel, Germany; 3 Klinikum Mittelbaden Baden-Baden Bühl, Balger Strasse 50, 76532 Baden-Baden Weststadt, Germany; 4 Hämatologisch-Onkologische Praxis, Mauerfeldchen 72, 52146 Würselen, Germany; 5 Gynäkologisches Zentrum Bonn, Friedenplatz 16, 53111 Bonn, Germany; 6 Hämatologisch-onkologische Tagesklinik, Achdorfer Weg 5, 84036 Landshut, Germany; 7 Klinikum Leverkusen gGmbH, Am Gesundheitspark 11, 51375 Leverkusen, Germany; 8 MediOnko-Institut GbR, Möllendorffstr. 52, 10367 Berlin, Germany; 9 St.-Johannes-Hospital, Johannesstr. 9-17, 44137 Dortmund, Germany; 10 iOMEDICO AG, Ellen-Gottlieb-Straße 19, 79106 Freiburg im Breisgau, Germany

**Keywords:** Advanced breast Cancer, Endocrine therapy, Combined chemo- and anti-Angiogenic therapy, Patient preference, Randomized, cross-over phase IV study

## Abstract

**Background:**

The objective of the IMPROVE study was patients’ preference for either endocrine-based therapy or combined chemo- and anti-angiogenic therapy in advanced HR-positive/HER2-negative breast cancer.

**Methods:**

In this randomized, cross-over phase IV study, 77 patients were recruited in 26 sites in Germany. Patients were randomized 1:1 to receive either capecitabine plus bevacizumab (Cap+Bev) as first-line therapy followed by cross-over to everolimus plus exemestane (Eve+Exe) as second-line therapy (Arm A) or the reverse sequence (Arm B). The primary endpoint was patients’ preference for either regimen, assessed by the Patient Preference Questionnaire 12 weeks after cross-over. Key secondary endpoints included progression-free survival (PFS), overall survival (OS), safety, and quality of life (QoL).

**Results:**

61.5% of patients preferred Cap+Bev (*p* = 0.1653), whereas 15.4% preferred Eve+Exe and 23.1% were indecisive. Physicians showed a similar tendency towards Cap+Bev (58.1%) as the preferred regimen versus Eve+Exe (32.3%). Median first-line PFS was longer for Cap+Bev than for Eve+Exe (11.1 months versus 3.5 months). Median second-line PFS was similar between Cap+Bev and Eve+Exe (3.6 months versus 3.7 months). Median OS was comparable between Arm A (28.8 months) and Arm B (24.7 months). 73.0% and 52.6% (first−/second-line, Cap+Bev) and 54.1% and 52.9% (first−/second-line, Eve+Exe) of patients experienced grade 3/4 TEAEs. No treatment-related deaths occurred. QoL and treatment satisfaction were not significantly different between arms or treatment lines.

**Conclusions:**

Patients tended to favor Cap+Bev over Eve+Exe, which was in line with physicians’ preference. Cap+Bev showed superior first-line PFS, while QoL was similar in both arms. No new safety signals were reported.

**Trial registration:**

EudraCT No: 2013–005329-22. Registered on 19 August 20

## Background

Breast cancer (BC) is the second-most common cancer worldwide. Approximately 20–30% of all patients treated with curative intent develop metastatic BC (MBC) [1]. Depending on subtype, systemic treatment of advanced BC (locally recurrent and inoperable or MBC) offers a wide range of options including endocrine therapy, immunochemotherapy, kinase inhibitors, radiation therapy, and supportive measures. For postmenopausal patients with advanced hormone receptor-positive/human epidermal growth factor receptor 2-negative (HR-positive/HER2-negative) BC various treatment options exist. Current international guidelines recommend endocrine therapy as the first-line treatment of choice in the vast majority of these patients. Chemotherapy may be considered as a reasonable alternative in patients for whom endocrine treatment is inappropriate including individuals presenting with acute visceral crisis and/or hormone-resistant tumors [2–4]. Up to now, there are limited data from clinical trials having directly compared chemotherapy and endocrine-based treatment in patients with MBC. A previous meta-analysis reported similar efficacy of both treatment strategies, however, the drugs utilized were outdated as per today’s standard of care [5].

Capecitabine (Cap) + bevacizumab (Avastin®, Bev) combination therapy is indicated for first-line treatment of adult patients with MBC for whom treatment with taxanes and/or anthracyclines is inappropriate [6–9]. Regarding endocrine therapy, the everolimus (Afinitor®, Eve) + exemestane (Aromasin®, Exe) combination is indicated in postmenopausal patients with advanced HR-positive, HER2-negative BC after recurrence or progression following treatment with non-steroidal aromatase inhibitors [10–13].

Assuming comparable efficacy between different therapeutic options, it is of utmost importance to identify the treatment with the least negative impact on patients’ quality of life (QoL). The IMPROVE study evaluated patients’ preference for either combined antihormonal therapy (everolimus + exemestane, Eve+Exe) or combined chemo- and anti-angiogenic therapy (capecitabine + bevacizumab, Cap+Bev) in postmenopausal patients with advanced HR-positive, HER2-negative BC and indication for first-line chemotherapy or endocrine therapy after failure of ≥1 standard non-steroidal aromatase inhibitor therapy.

## Methods

## Patients

Eligible patients were aged ≥18 years, postmenopausal, diagnosed with HR-positive, HER2-negative advanced BC (locally recurrent and inoperable or MBC), with indication for first-line chemotherapy or endocrine therapy after failure of ≥1 non-steroidal aromatase inhibitor therapy, no indication for treatment with taxanes and/or anthracyclines, measurable or non-measurable disease according to RECIST v1.1 [14], adequate bone marrow and organ function as per current Summary of Product Characteristics (SmPC) of respective study drug [8, 9, 12, 13], ECOG performance status ≤2, and fluency in German. Key exclusion criteria included prior palliative cytotoxic chemotherapy, prior treatment with mTOR (mammalian target of rapamycin)-inhibitors (prior treatment with exemestane was permitted), symptomatic visceral metastases, unstable skeletal metastases, medically uncontrolled cardiovascular diseases or diabetes mellitus, and known dihydropyrimidine dehydrogenase deficiency.

## Study design and endpoints

**Fig. 1:**
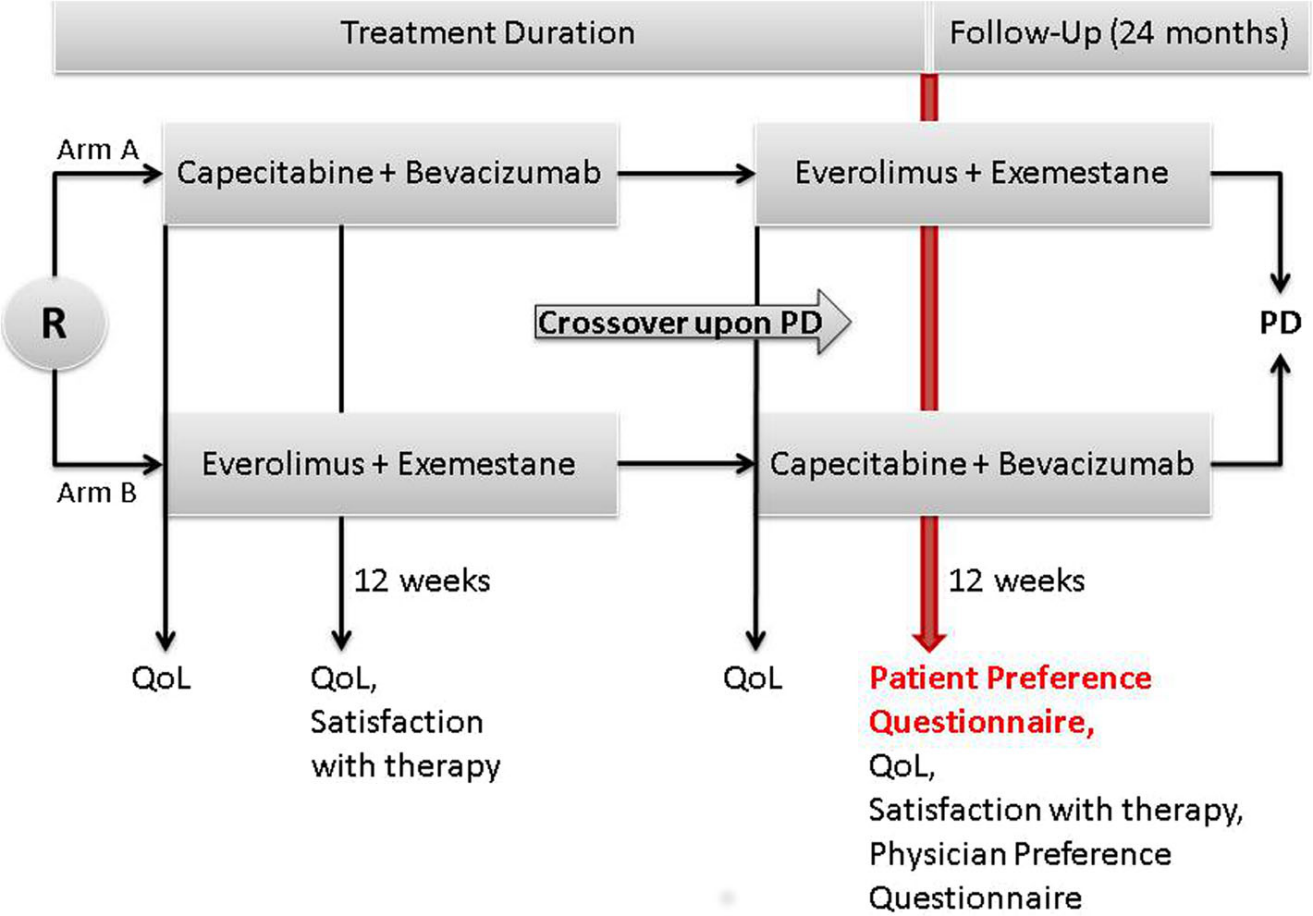
Overall Study Design. Duration of follow-up: ≥24 months, until death or end of study. R = randomization; PD = progressive disease; QoL = quality of life

This open-label, randomized, controlled, multicenter, two-arm, cross-over phase IV study (EudraCT No 2013–005329-22) randomly assigned patients (1:1) to receive either Cap+Bev first-line therapy during the first treatment phase (TP) followed by cross-over to Eve+Exe as second-line therapy during the second TP (Arm A) or the reverse sequence (Arm B; Fig. 1). For allocation of patients, a computer-generated randomization list was used to automatically allocate patients in the electronic Case Report Form by using an integrated randomization tool within the Electronic Data Capture system. Patients were stratified by (i) visceral metastases versus non-visceral metastases, (ii) prior (neo) adjuvant treatment (anthracycline and/or taxane; yes versus no), (iii) number of prior palliative anti-hormonal therapies (0–1 versus > 1) and (iv) disease-free interval (DFI; ≤2 years versus > 2 years) by using a permuted block randomization with block lengths of two and four. DFI was defined as the time from first R0 resection until first local relapse or occurrence of distant metastases, whichever occurred first. The block sizes were not disclosed to ensure concealment. Patients were treated in each TP until progression, intolerable toxicity, or withdrawal of consent. Each TP was separated by a washout phase (7–28 days). Dosing and administration of the study drugs are detailed in Table 1.

**Table 1:** Dose and mode of administration of the study drugs^a^

Study drug	Dose and mode of administration
Capecitabine	1000 mg/m^2^ per os twice daily as combined 150 mg and 500 mg tablets on days 1 to 14 of each 21-day cycle, followed by a 7-day resting period (i.e. off-treatment)
Bevacizumab	15 mg/kg intravenously once every 3 weeks (i.e. 5 mg/kg/week dose equivalent)
Everolimus	10 mg per os once daily of each 21-day cycle
Exemestane	25 mg per os once daily of each 21-day cycle

^a^Dose and mode of administration of the study drugs were in accordance with current SmPC of respective study drug. Study drugs were available upon prescription by respective treating physician. A cycle was defined as 21 days of study treatment

The primary endpoint was patients’ preference for either Cap+Bev or Eve+Exe combination therapy, assessed by questionnaire 12 weeks after cross over. Key secondary endpoints included progression-free survival (PFS), overall survival (OS), treatment satisfaction, QoL, and safety.

### Questionnaires

Patients’ treatment preference was assessed by the Patient Preference Questionnaire (PPQ) 12 weeks after cross over. Patients having discontinued therapy after < 12 weeks of treatment were asked to complete the PPQ within 2 weeks of treatment discontinuation. Patients were asked in the PPQ for their treatment preference (Cap+Bev, Eve+Exe, or no preference) and reason for their preference. Similarly, physicians were asked for their treatment preference (case-based), assessed by the Physician Preference Questionnaire. Patients were asked to complete the Treatment Satisfaction Questionnaire in week 12 of each TP. Additionally, patients were asked to complete QoL questionnaires in each TP (baseline and at week 12) including the European Organisation for Research and Treatment of Cancer (EORTC) quality of life questionnaire (QLQ)-C30 questionnaire [15].

### Tumor assessment and adverse events

Tumor evaluation according to RECIST v1.1 was to be performed by the investigator at screening, every 12 weeks and at end of each TP, and every 6 months during follow-up (FU; ≥24 months or until death). During the FU, patients were also assessed for survival status. Any treatment-emergent adverse event (TEAE) and toxicity were to be recorded from day of first administration of study treatment until 30 days after end of treatment in each TP. TEAEs were graded by the investigator according to NCI CTCAE v4.03 [16].

### Statistical analysis

The initial null hypothesis stated that there is no difference in patients’ preference for either regimen assuming that 80% of patients do have a preference, whereas 20% cannot decide. The study was designed to control the α error rate at 0.05 with a power of 80%. Required sample size was estimated to 192 patients assuming a drop-out rate at 35%. The assumptions for the null hypotheses were chosen according to the PISCES trial [17].

The intent-to-treat (ITT) population comprised all patients to whom study treatment had been assigned by randomization. The modified ITT (mITT) population was used to assess the primary endpoint and was predefined as all patients who had received both first-line and second-line therapy for ≥12 weeks per line or less for other reasons than progressive disease (PD), crossed over to second-line therapy within 12 weeks of termination of first-line therapy, and answered the preference question. Additionally, the primary endpoint was assessed in a post hoc analysis by using the per-protocol (PP) population including all patients who had received ≥1 dose of study medication in first-line and second-line therapy and answered the preference question. The safety set (SAF) included all patients who had received ≥1 dose of study medication.

To analyze the primary endpoint and to test the significance of difference in patient preference between the regimens, a Chi-square test was used to compare the actual preference against the null hypothesis of no difference in patient preference taking into account the proportion of indifferent patients. For the final analysis, the null hypothesis was adopted for the actually observed proportion of indifferent patients. By this means, the actual null hypothesis for the primary analysis was 38.45% prefer Cap+Bev, 38.45% prefer Eve+Exe, and 23.1% are indifferent about their preference. Reasons for preference were analyzed descriptively.

Difference in overall treatment satisfaction was analyzed by using an asymptotic chi-square test (satisfied versus not satisfied).

The EORTC QLQ-C30 questionnaire was analyzed descriptively. Differences in scores were evaluated by a t-test.

PFS and OS were estimated by using the Kaplan-Meier method [18].

The primary endpoint was further evaluated by age (< 60 years vs. ≥60 years) in the mITT, ITT and PP populations. However, further subgroup and explorative analyses *pre-planned* for the primary endpoint were not performed due to low number of available observations (mITT; *N* = 13).

## Results

This study was initially planned to enroll 192 patients. Due to emergence of new treatment options and low recruitment rate, the recruitment was stopped after 77 patients had been randomized. The study was prematurely terminated in September 2017.

### Disposition of patients

**Fig. 2:**
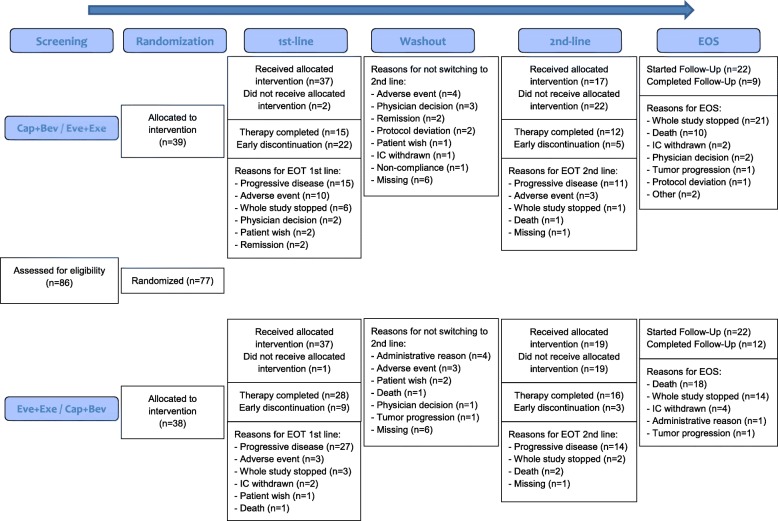
CONSORT Flow Diagram. The reason “Adverse event” includes both inacceptable toxicity and (serious) adverse event. A therapy was considered completed if the reason for end of treatment was either progressive disease or death. Follow-up was considered completed if the follow-up was started and the reason for end of study was death. For two patients, “death” was documented as the reason for end of treatment in second-line therapy, although the death occurred later than 30 days after end of treatment (safety follow-up period). EOT = end of treatment; EOS = end of study; IC = informed consent

Seventy-seven patients (ITT population) were recruited from October 2014 through April 2017 in 26 sites in Germany (Fig. 2); 39 patients were randomized to Arm A (Cap+Bev / Eve+Exe) and 38 patients to Arm B (Eve+Exe / Cap+Bev). In Arm A, 37 of the 39 randomized patients received the allocated first-line therapy. Of these, 17 patients crossed over to second-line therapy. In Arm B, 37 of the 38 randomized patients received the allocated first-line therapy. Of these, 19 patients crossed over to second-line therapy. Overall, 44 patients started the FU, which was planned to last ≥24 months or until death (FU completed: *n* = 21).

The mITT population comprised 13 patients (Arm A: *N* = 5; Arm B: *N* = 8) and the PP population 31 patients (Arm A: *N* = 13; Arm B: *N* = 18). The SAF population was used to analyze TEAEs in first-line (Arm A: *N* = 37; Arm B: *N* = 37) and second-line therapy (Arm A: *N* = 17; Arm B: *N* = 19).

### Patient characteristics at baseline

All patients had HR-positive, HER2-negative BC. Baseline patient demographics, clinical characteristics, and treatment history in the randomized ITT population were balanced between study arms (Table 2).

**Table 2:** Patient characteristics at baseline (ITT population)

	Arm A (Cap+Bev / Eve+Exe) (*N* = 39)	Arm B (Eve+Exe / Cap+Bev) (*N* = 38)
Age [years], median (range)	64.4 (47.0–83.6)	65.9 (49.8–86.0)
Ethnicity, n (%)		
Caucasian	38 (97.4%)	37 (97.4%)
Asian	1 (2.6%)	1 (2.6%)
BMI [kg/m^2^], median (quartiles)	25.6 (22.5–28.4)	24.9 (22.7–31.1)
ECOG Performance Status, n (%)		
0	19 (48.7%)	17 (44.7%)
1	19 (48.7%)	20 (52.6%)
2	1 (2.6%)	1 (2.6%)
Menopausal status, n (%)		
Postmenopausal	39 (100%)	38 (100%)
Concomitant diseases, n (%)		
Yes	33 (84.6%)	35 (92.1%)
No	6 (15.4%)	3 (7.9%)
Visceral disease or local relapse, n (%)	27 (69.2%)	26 (68.4%)
Non-visceral disease, n (%)	12 (30.8%)	12 (31.6%)
Metastatic sites^a^, n (%)		
Bone	28 (71.8%)	29 (76.3%)
Liver	15 (38.5%)	16 (42.1%)
Lung	8 (20.5%)	10 (26.3%)
Histology of tumor, n (%)		
Invasive ductal	27 (69.2%)	24 (63.2%)
Invasive lobular	7 (17.9%)	12 (31.6%)
Inflammatory cancer	1 (2.6%)	0
Not otherwise specified	4 (10.3%)	2 (5.3%)
DFI^b^ [years], median (quartiles)	4.7 (1.4–10.9)	5.0 (1.3–8.2)
Time since initial diagnosis [years], median (quartiles)	9.6 (3.6–13.0)	6.6 (2.5–10.0)
Time since first relapse^c^ [years], median (quartiles)	8.4 (0.6–23.4)	5.1 (1.5–15.1)
Any prior adjuvant chemotherapy, n (%)	22 (56.4%)	28 (73.7%)
Prior adjuvant taxane and/or anthracycline therapy, n (%)	22 (56.4%)	26 (68.4%)
Number of prior palliative endocrine therapies, n (%)		
1	10 (25.6%)	13 (34.2%)
2	11 (28.2%)	10 (26.3%)
3	1 (2.6%)	0

^a^Multiple answers per patient were possible. ^b^DFI was defined as the time from first R0 resection until first local relapse or occurrence of distant metastases, whichever occurred first. ^c^Includes both local breast cancer recurrence and distant metastases. BMI = body mass index; ECOG = Eastern Cooperative Oncology Group; DFI = disease-free interval

### Patients’ preference

Overall, 61.5% of patients reported Cap+Bev as their preferred regimen, though not statistically significant (mITT, *p* = 0.1653, Table 3). In Arm A and Arm B, 40% and 75% of patients reported Cap+Bev as their preferred therapy versus Eve+Exe (Arm A: 20.0%; Arm B: 12.5%), respectively. Overall, a similar tendency towards Cap+Bev as the preferred regimen was observed in the PP and ITT populations (Table 3). Notably, 10 of the 13 patients preferring Cap+Bev were found in Arm B.

**Table 3:** Rates of patients’ preference^a^ (mITT/ITT/PP population)

mITT	Arm A (N = 5)		Arm B (N = 8)		Total (*N* = 13)	
	*n* (%)	95%-CI	*n* (%)	95%-CI	*n* (%)	95%-CI
Cap+Bev	2 (40.0%)	[5.3, 85.3]	6 (75.0%)	[34.9, 96.8]	8 (61.5%)	[31.6, 86.1]
Eve+Exe	1 (20.0%)	[0.5, 71.6]	1 (12.5%)	[0.3, 52.7]	2 (15.4%)	[1.9, 45.4]
I cannot decide	2 (40.0%)	[5.3, 85.3]	1 (12.5%)	[0.3, 52.7]	3 (23.1%)	[5.0, 53.8]
p-value (Chi-square)					0.1653	
ITT	Arm A (*N* = 39)		Arm B (*N* = 38)		Total (*N* = 77)	
	n (%)	95%-CI	n (%)	95%-CI	n (%)	95%-CI
Cap+Bev	3 (7.7%)	[1.6, 20.9]	10 (26.3%)	[13.4, 43.1]	13 (16.9%)	[9.3, 27.1]
Eve+Exe	5 (12.8%)	[4.3, 27.4]	2 (5.3%)	[0.6, 17.7]	7 (9.1%)	[3.7, 17.8]
I cannot decide	3 (7.7%)	[1.6, 20.9]	4 (10.5%)	[2.9, 24.8]	7 (9.1%)	[3.7, 17.8]
Not evaluable	2 (5.1%)	[0.6, 17.3]	2 (5.3%)	[0.6, 17.7]	4 (5.2%)	[1.4, 12.8]
Item / questionnaire not answered	4 (10.3%)	[2.9, 24.2]	1 (2.6%)	[0.1, 13.8]	5 (6.5%)	[2.1, 14.5]
No second-line therapy	22 (56.4%)	[39.6, 72.2]	19 (50.0%)	[33.4, 66.6]	41 (53.2%)	[41.5, 64.7]
PP	Arm A (*N* = 13)		Arm B (*N* = 18)		Total (*N* = 31)	
	*n* (%)	95%-CI	*n* (%)	95%-CI	*n* (%)	95%-CI
Cap+Bev	3 (23.1%)	[5.0, 53.8]	10 (55.6%)	[30.8, 78.5]	13 (41.9%)	[24.5, 60.9]
Eve+Exe	5 (38.5%)	[13.9, 68.4]	2 (11.1%)	[1.4, 34.7]	7 (22.6%)	[9.6, 41.1]
I cannot decide	3 (23.1%)	[5.0, 53.8]	4 (22.2%)	[6.4, 47.6]	7 (22.6%)	[9.6, 41.1]
Not evaluable	2 (15.4%)	[1.9, 45.4]	2 (11.1%)	[1.4, 34.7]	4 (12.9%)	[3.6, 29.8]

^a^Patient’s treatment preference was evaluated after 12 weeks of second-line treatment, assessed by the Patient Preference Questionnaire. Confidence interval was calculated using the Clopper-Pearson formula. Due to small n, the p-value of the asymptotic chi-square test may not be valid. The *p*-value of the corresponding exact test was 0.1666. Not evaluable: Patients selected more than one possible answer. mITT = modified ITT; ITT = intent-to-treat; PP = per-protocol; CI = confidence interval

The main reason for preference was improved QoL reported both among patients (ITT population) having preferred Cap+Bev (*n* = 9; 69.2%) or Eve+Exe (*n* = 3; 42.9%).

Patient’s preference was further evaluated by age (< 60 years vs. ≥60 years) in the mITT, ITT and PP populations. Overall, a similar pattern towards a preference for Cap+Bev over Eve+Exe was observed in both age groups in all three analytical populations (data not shown).

### Physician’s preference

Overall, physicians rated in 46.2% and 30.8% of their cases Cap+Bev and Eve+Exe as their preferred regimen (mITT, Table 4), respectively. A similar pattern was observed in the PP and ITT populations (Table 4).

**Table 4:** Rates of physician’s preference^a^ (mITT/ITT/PP population)

mITT	Arm A (*N* = 5)		Arm B (*N* = 8)		Total (*N* = 13)	
	*n* (%)	95%-CI	*n* (%)	95%-CI	*n* (%)	95%-CI
Cap+Bev	3 (60.0%)	[14.7, 94.7]	3 (37.5%)	[8.5, 75.5]	6 (46.2%)	[19.2, 74.9]
Eve+Exe	2 (40.0%)	[5.3, 85.3]	2 (25.0%)	[3.2, 65.1]	4 (30.8%)	[9.1, 61.4]
I cannot decide	0		3 (37.5%)	[8.5, 75.5]	3 (23.1%)	[5.0, 53.8]
ITT	Arm A (N = 39)		Arm B (N = 38)		Total (N = 77)	
	n (%)	95%-CI	n (%)	95%-CI	n (%)	95%-CI
Cap+Bev	10 (25.6%)	[13.0, 42.1]	10 (26.3%)	[13.4, 43.1]	20 (26.0%)	[16.6, 37.2]
Eve+Exe	5 (12.8%)	[4.3, 27.4]	5 (13.2%)	[4.4, 28.1]	10 (13.0%)	[6.4, 22.6]
I cannot decide	2 (5.1%)	[0.6, 17.3]	4 (10.5%)	[2.9, 24.8]	6 (7.8%)	[2.9, 16.2]
PP	Arm A (N = 13)		Arm B (N = 18)		Total (N = 31)	
	n (%)	95%-CI	n (%)	95%-CI	n (%)	95%-CI
Cap+Bev	8 (61.5%)	[31.6, 86.1]	10 (55.6%)	[30.8, 78.5]	18 (58.1%)	[39.1, 75.5]
Eve+Exe	5 (38.5%)	[13.9, 68.4]	5 (27.8%)	[9.7, 53.5]	10 (32.3%)	[16.7, 51.4]
I cannot decide	0		3 (16.7%)	[3.6, 41.4]	3 (9.7%)	[2.0, 25.8]

^a^Physician’s preference was evaluated after 12 weeks of second-line treatment, assessed by the Physician Preference Questionnaire. Confidence interval was calculated using the Clopper-Pearson formula. Discrepancies between the sum of patients in the ITT population and the total n reported are due to patients, who did not cross over to the second-line therapy. mITT = modified ITT; ITT = intent-to-treat; PP = per-protocol; CI = confidence interval

### Treatment satisfaction

The majority of the patients in the ITT population in both arms were satisfied with first-line therapy (Arm A: 76.9%; Arm B: 63.2%) and second-line therapy (Arm A: 58.8%; Arm B: 57.9%). There was no major difference between arms either in first-line therapy or in second-line therapy (Table 5).

**Table 5:** Overall treatment satisfaction^a^ (ITT population)

	First-line therapy		Second-line therapy	
	Arm A (*N* = 39)	Arm B (*N* = 38)	Arm A (*N* = 17)	Arm B (*N* = 19)
Satisfied	30 (76.9%)	24 (63.2%)	10 (58.8%)	11 (57.9%)
Not satisfied	5 (12.8%)	7 (18.4%)	3 (17.6%)	5 (26.3%)
Missing		2 (5.3%)	1 (5.9%)	2 (10.5%)
p-value	0.3832		0.6243	

^a^Treatment satisfaction was evaluated in week 12 of each treatment phase, assessed by the Treatment Satisfaction Questionnaire. Second-line: Due to small n, the *p*-value of the asymptotic chi-square test may not be valid. The p-value of the corresponding exact test was 0.6968. Missing: Item was not answered or not evaluable. Discrepancies between the sum of answers reported and the total n are due to patients, who did not answer the whole questionnaire

### Global health status (EORTC-QLQ-C30)

**Fig. 3:**
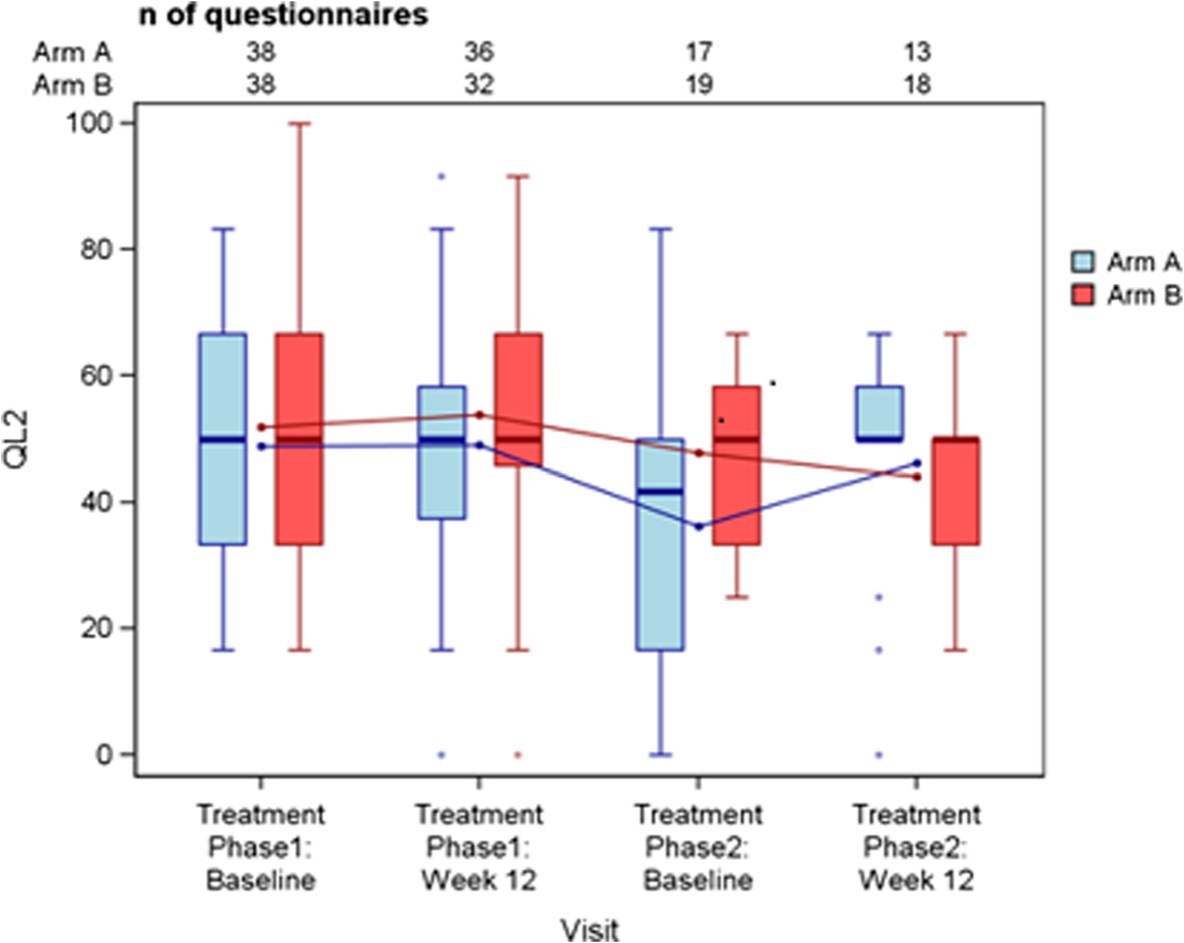
EORTC-QLQ-C30 – Global Health Status. Depicted are box-and-whisker plots showing the scores of global health status in the ITT population across Arm A (Cap+Bev/Eve+Exe) and Arm B (Eve+Exe/Cap+Bev) at baseline and at week 12 in each treatment phase. The scale ranges from 0 to a maximum of 100. The horizontal solid line within each box shows the median. The line graphs display the mean Quality of Life over time per study arm. The lines extending vertically from the boxes (whiskers) indicate the variability outside the upper and lower quartiles. The whiskers span 1.5 x IQR (interquartile range, i.e. the difference between the quartiles). The individual small circles represent the outliers. EORTC QLQ-C30 = European Organisation for Research and Treatment of Cancer Quality of Life Questionnaire-C30 (30-item core module); n = number; QL2 = Quality of life

Median global health score was 50.0 during the first TP across both arms both at baseline and at week 12. There was no significant difference in the mean scores of global health status between arms in either TP (ITT, Fig. 3).

### Progression-free survival

**Fig. 4:**
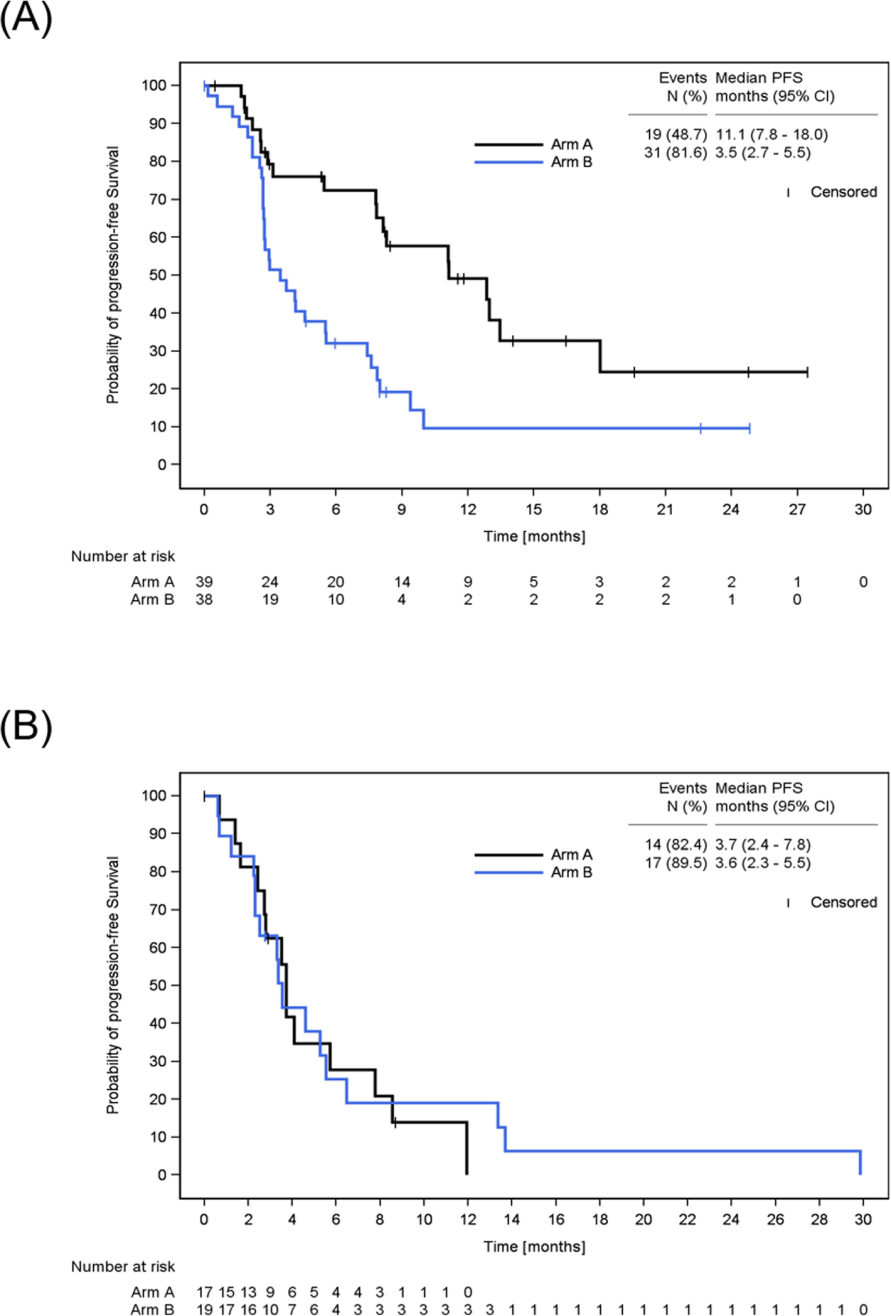
Kaplan-Meier Estimate of Progression-Free Survival. Displayed are first-line (A) and second-line (B) progression-free survival in Arm A (Cap+Bev/Eve+Exe) and Arm B (Eve+Exe/Cap+Bev) in the ITT population. First-line and second-line progression-free survival were defined as the time from start of first-line / second-line therapy to progression or death due to any cause prior to start of new therapy, respectively. Patients without progression or death were censored at the date of last tumor evaluation in first-line / second-line therapy, respectively. N = number; PFS = progression-free survival; CI = confidence interval

Median first-line PFS was longer (*p* = 0.0008) in Arm A (11.1 months) than in Arm B (3.5 months, Fig. 4a). 51.3% of the patients in Arm A were censored for first-line PFS. The estimated 3-month first-line PFS rate was 79.3% (95% CI: 61.4–89.6) in Arm A and 51.4% (95% CI: 34.4–65.9) in Arm B. There was no major difference (*p* = 0.8345) in median second-line PFS between Arm A (3.7 months) and Arm B (3.6 months, Fig. 4b). The estimated 3-month second-line PFS rate was similar between Arm A (62.5%; 95% CI: 34.9–81.1) and Arm B (63.2%; 95% CI: 37.9–80.4).

### Overall survival

**Fig. 5:**
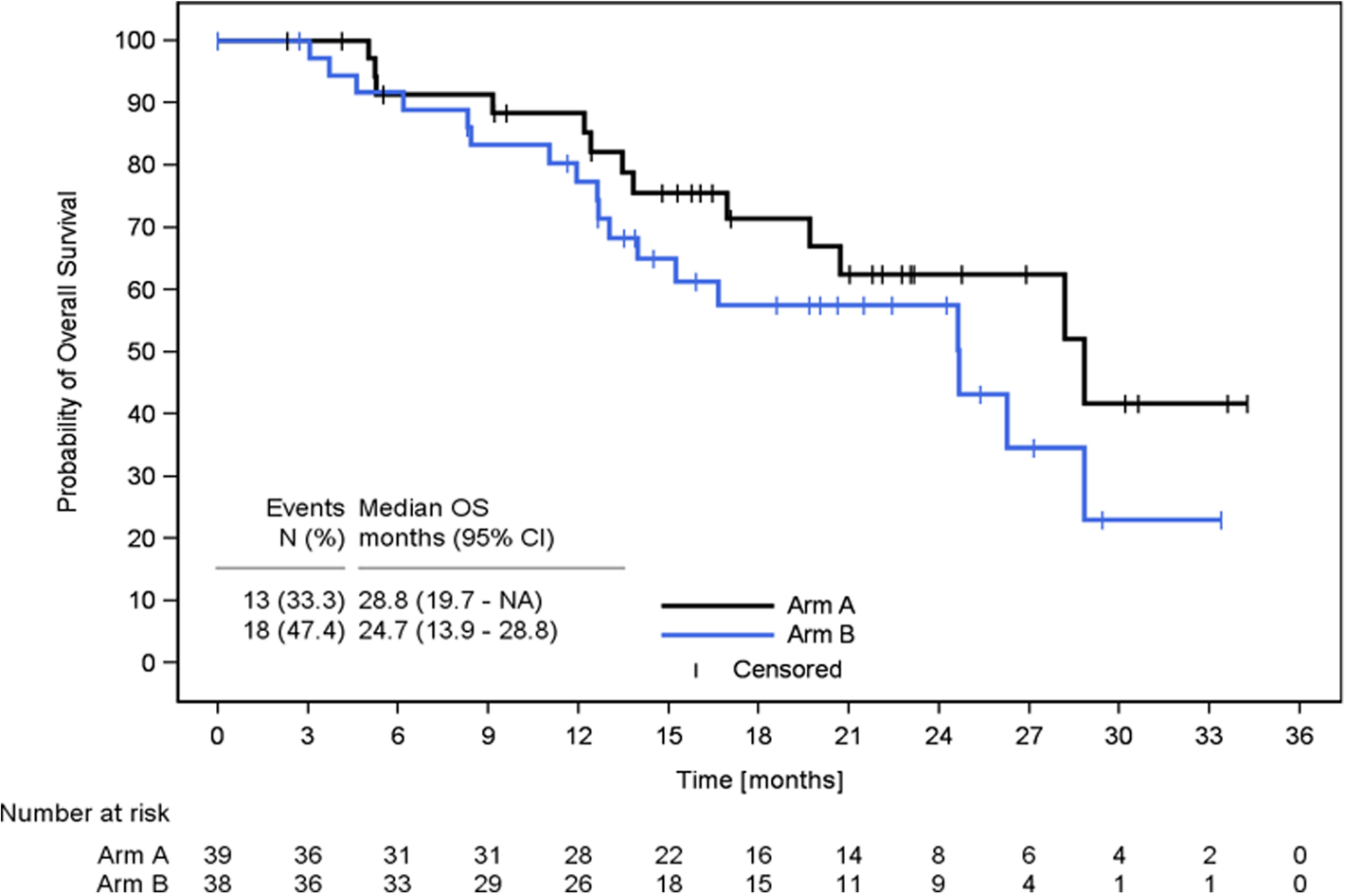
Kaplan-Meier Estimate of Overall Survival. Displayed is overall survival in Arm A (Cap+Bev/Eve+Exe) and Arm B (Eve+Exe/Cap+Bev) in the ITT population. Overall survival was defined as the time from start of first-line therapy to death due to any cause. Patients without documented date of death at the end of study were censored at the last date known to be alive. N = number; OS = overall survival; CI = confidence interval; NA = not reached

Thirty-one patients (Arm A: *n* = 13; Arm B: *n* = 18) died during the course of the study. There was no major difference (*p* = 0.2088) in median OS between Arm A (28.8 months) and Arm B (24.7 months, Fig. 5).

### Safety

Median duration of Cap+Bev therapy (first-line, 6.4 months; second-line, 4.0 months) was longer in both TPs as compared to Eve+Exe therapy (first-line, 3.4 months; second-line, 2.7 months).

Four fatal serious TEAEs (Arm A; n = 1; Arm B: *n* = 3) due to PD were reported during the course of the study; none were attributable to any study drug. 73.0% and 52.6% of the patients in Cap+Bev first-line and second-line therapy experienced TEAEs of CTCAE grade 3/4, respectively. 54.1% and 52.9% of the patients in Eve+Exe first-line and second-line therapy experienced TEAEs of CTCAE grade 3/4, respectively. Most common TEAEs of CTCAE grade 3/4 were observed in Arm A including hand-foot syndrome (18.9%) and hypertension (13.5%) during Cap+Bev first-line therapy, and pain (11.8%) and pneumonia (11.8%) during Eve+Exe second-line therapy (Table 6).

**Table 6:** Treatment-emergent adverse events (> 10% any grade; SAF population)

	Arm A				Arm B			
	First-line (Cap+Bev) *N* = 37		Second-line (Eve+Exe) *N* = 17		First-line (Eve+Exe) *N* = 37		Second-line (Cap+Bev) *N* = 19	
	Any grade	Grade 3/4	Any grade	Grade 3/4	Any grade	Grade 3/4	Any grade	Grade 3/4
Adverse event, *n* (%)								
Anemia	1 (2.7%)		4 (23.5%)		5 (13.5%)			
Arthralgia	5 (13.5%)				4 (10.8%)		1 (5.3%)	
Bone pain	3 (8.1%)				6 (16.2%)			
Cough	5 (13.5%)		3 (17.6%)		6 (16.2%)		2 (10.5%)	
Decreased appetite	6 (16.2%)		1 (5.9%)		5 (13.5%)			
Diarrhea	12 (32.4%)		2 (11.8%)		11 (29.7%)		5 (26.3%)	
Dyspnea	6 (16.2%)		3 (17.6%)		4 (10.8%)		4 (21.1%)	
Fatigue	13 (35.1%)		3 (17.6%)		6 (16.2%)		2 (10.5%)	
Hand-Foot Syndrome	21 (56.8%)	7 (18.9%)			1 (2.7%)		6 (31.6%)	2 (10.5%)
Headache	6 (16.2%)				6 (16.2%)			
Hypertension	11 (29.7%)	5 (13.5%)			2 (5.4%)		3 (15.8%)	
Mucosal inflammation	4 (10.8%)		2 (11.8%)		7 (18.9%)			
Nausea	15 (40.5%)		1 (5.9%)		9 (24.3%)		5 (26.3%)	
Edema peripheral	2 (5.4%)				6 (16.2%)		2 (10.5%)	
Pain	2 (5.4%)		2 (11.8%)	2 (11.8%)	1 (2.7%)			
Pleural effusion	2 (5.4%)				2 (5.4%)		3 (15.8%)	
Pneumonia				2 (11.8%)	3 (8.1%)			
PSN	6 (16.2%)				1 (2.7%)		2 (10.5%)	
Stomatitis	9 (24.3%)		1 (5.9%)		5 (13.5%)		1 (5.3%)	

Percentages in second-line therapy refer to the number of patients, who started second-line therapy. Time range: from first administration of study treatment until 30 days after end of treatment in each treatment line. Unrelated TEAEs starting within 30 days of end of first-line therapy and after start of second-line therapy were considered treatment-emergent in both treatment lines in case second-line therapy had started within 30 days of end of first-line therapy. MedDRA v20.0. PSN = Peripheral sensory neuropathy

## Discussion

Current guidelines unequivocally indicate endocrine therapy as the preferred systemic treatment in the vast majority of patients with advanced HR-positive, HER2-negative BC [2–4]. However, this is in contrast to treatment reality; 43% of these patients are primarily subjected to first-line chemotherapy with > 80% receiving first-line chemotherapy when aged ≤50 years [19]. The reason for this non-adherence remains unclear at present.

Knowing patients’ treatment preference is paramount in patient care. There is a renewed interest in implementation of a shared decision-making process between patients and healthcare, ultimately enhancing treatment quality [20, 21]. Notably, it has been proven that patients with BC (16%) value the efficacy less than the side-effects [22] and are willing to make trade-offs between side-effects and different regimens [23].

Although prematurely terminated, to our knowledge the IMPROVE study is the first randomized study having evaluated patients’ preference for either combined chemo- and anti-angiogenic therapy (Cap+Bev) or combined endocrine therapy (Eve+Exe) in postmenopausal patients with advanced HR-positive, HER2-negative BC. Furthermore, IMPROVE is the first study to date having compared the efficacy outcome of endocrine-based therapy with an anti-angiogenic-based chemotherapy approach.

In this study, patients tended to favor Cap+Bev over Eve+Exe (mITT), which was in line with physicians’ preference. The potential influence from the treating physician should be taken into account. However, the primary endpoint could not be adequately assessed due to low number of observations owing to premature termination of the study and low number of PPQs as a high number of patients did not cross over to second-line therapy. Therefore, the data are of limited interpretability and further conclusions cannot be made. Nevertheless, despite the low number of patients in the mITT population, the data provide important and valuable novel information on patient preference regarding endocrine-based therapy versus anti-angiogenic-based chemotherapy in advanced BC. A similar tendency towards Cap+Bev as patients’ and physicians’ preferred regimen was observed in the PP population. It is intriguing to also look at the ITT population; overall, patients tended to prefer Cap+Bev over Eve+Exe. The main reason for deciding for Cap+Bev was improved QoL. However, patient-reported QoL was similar in both arms bearing in mind the large variation in QoL scores across arms and TPs. Keeping in mind the small number of evaluable questionnaires in the ITT population, it was nevertheless surprising to see Cap+Bev as the preferred regimen, despite the higher percentage of first-line grade 3/4 TEAEs observed in Arm A, possibly attributable to the differences in treatment duration between regimens.

Median first-line PFS was longer in Arm A than in Arm B. The PFS of only 3.5 months in Arm B was unexpected when comparing with the BOLERO-2 trial [10], which reported a superior median PFS of 6.9 months/10.6 months (local/central assessment) in the Eve+Exe arm. Possible reasons for the short PFS in our study include the low number of patients in each arm, the slightly shorter DFI and more prior treatments in Arm B versus Arm A, the higher percentage of comorbidities observed in Arm B (92.1%) compared to Arm A (84.6%) and a higher percentage of patients in Arm B (68.4%) with prior adjuvant treatment with anthracycline/taxane versus Arm A (56.4%), possibly reflecting a higher risk profile at initial diagnosis in Arm B. In the BOLERO-6 trial, a median PFS of 8.4 months was reported for Eve+Exe combination therapy versus median 9.6 months for Cap monotherapy [24]; although not directly comparable, these results point towards the data in our study. Due to a high number of censored cases in the present study, the OS data are not further interpretable.

The safety profile in this study was consistent with current SmPC of respective drug [8, 9, 12, 13].

## Conclusion

Although prematurely terminated, to our knowledge the IMPROVE study is the first randomized study having evaluated patients’ preference for either combined chemo- and anti-angiogenic therapy (Cap+Bev) or combined endocrine therapy (Eve+Exe) in postmenopausal patients with advanced HR-positive, HER2-negative BC. There was a tendency to favor Cap+Bev over Eve+Exe, which was in line with physicians’ preference. The efficacy of Cap+Bev proved to be superior in terms of first-line PFS. Patient-reported QoL was similar in both arms. No new safety signals were reported.

## Data Availability

Clinical data were documented in electronic Case Report Forms (eCRFs; *iostudy office edc*, iOMEDICO) and are the property of iOMEDICO. The data are not publicly available due to them containing information that could compromise patient and/or research participant privacy / consent.

## Abbreviations

BC: Breast cancer
Bev: Bevacizumab
Cap: Capecitabine
CTCAE: Common Terminology Criteria for Adverse Events
DFI: Disease-free interval
ECOG: Eastern Cooperative Oncology Group
EORTC: European Organisation for Research and Treatment of Cancer
Eve: Everolimus
Exe: Exemestane
FU: Follow up
HER2: Human epidermal growth factor receptor 2
HR: Hormone receptor
ITT: Intent-to-treat
MBC: Metastatic BC
mITT: Modified ITT
mTOR: Mammalian target of rapamycin
NCI: National Cancer Institute
OS: Overall survival
PD: Progressive disease
PFS: Progression-free survival
PP: Per-protocol
PPQ: Patient preference questionnaire
QLQ: Quality of life questionnaire
QoL: Quality of life
RECIST: Response Evaluation Criteria In Solid Tumors
SAF: Safety set
SmPC: Summary of Product Characteristics
TEAE: Treatment-emergent adverse event
TP: Treatment phase
